# The Effects of Laser Marking and Symbol Etching on the Fatigue Life of Medical Devices

**DOI:** 10.1155/2013/570354

**Published:** 2013-04-07

**Authors:** P. J. Ogrodnik, C. I. Moorcroft, P. Wardle

**Affiliations:** Staffordshire University, Stafford ST18 0AD, UK

## Abstract

This paper examines the question;“ does permanent laser marking affect the mechanical performance of a metallic medical component?” The literature review revealed the surprising fact that very little has been presented or studied even though intuition suggests that its effect could be detrimental to a component's fatigue life. A brief investigation of laser marking suggests that defects greater than 25 *μ*m are possible. A theoretical investigation further suggests that this is unlikely to cause issues with relation to fast fracture but is highly likely to cause fatigue life issues. An experimental investigation confirmed that laser marking reduced the fatigue life of a component. This combination of lines of evidence suggests, strongly, that positioning of laser marking is highly critical and should not be left to chance. It is further suggested that medical device designers, especially those related to orthopaedic implants, should consider the position of laser marking in the design process. They should ensure that it is in an area of low stress amplitude. They should also ensure that they investigate worst-case scenarios when considering the stress environment; this, however, may not be straightforward.

## 1. Introduction

It is compulsory to have medical devices permanently marked. Commonly, this information is the part number for the component, the manufacturer's trademark, and the CE symbol (in Europe) and a lot number; to ensure legibility, the size of the lettering is further governed. This means that the positioning of the marking can be arbitrary and may be positioned purely because of available free space away from holes, changes in profile, and so forth. For metallic components, this marking is commonly produced using laser techniques. Most commonly, the laser is used to etch the surface of the component producing a defect whose surface finish is different from the rest of the body, hence making a discernible area. Controlling the position of the laser using CNC techniques can produce letters and graphics. The power and duration of the laser dictate the depth of the etching. The technical details [1] for a specific laser marking device (designed to minimize fatigue issues in safety critical components) state the following:

*“Laser etching markers work by focusing energy directly on the surface to be marked. The heat generated by the beam actually alters the surface of the part or vaporizes surface material… in other metals (than steel), the surface is etched when material is removed by high temperature vaporization… it modifies the metal alloying and etches the surface in a way that degrades the part's strength and can lead to fatigue or stress corrosion crack failure.” *

This is not a bold statement; it is highlighted in several commercial publications. Rosecrans [2], for example, investigated the effect of laser marking on typical materials used in components for the space shuttle. Rosecrans found that the effects on fatigue life were not predictable; in some materials, the fatigue life was markedly reduced, and in others, there was no effect. In 2007, Grivas et al. [3] presented a case report following the failure of a total hip arthroplasty; in this paper, they highlighted the laser marking as a potential source for the fatigue failure of the component. However, this was a forensic study after failure. Prior to this paper [4] had presented an examination of the effect of laser marking on stress corrosion resistance of stainless steels. They stated that the use of a nano-second-based system has a marked deleterious effect on the material's performance. They further highlighted that little research had been conducted in this area which is rather surprising considering the implications. Although one may infer the effects of laser marking from classic fatigue theory, it is surprising that there is little research verifying or disproving the inferences.

Modern implants are designed to last for many years. In the case of an orthopaedic implant in a leg, this could mean 3 million loading cycles per year [5], and hence, it must be designed to be able to withstand fatigue failure. For example, a plate used to treat a plateau fracture of the tibia may be required to withstand cyclic loading for up to 2 years (before the fracture has fully healed), and hence it may be required to withstand 6 million cycles. It is, therefore, in a category that requires fatigue failures to be analysed and minimised. To this extent, the surface finish of these components is such that they are, to all intents and purposes, defect-free. Indeed, it is common for these components to have a virtual mirror finish (a finish commonplace in motorsports specifically to minimize fatigue failures). Unfortunately, as described earlier, they also require permanent marking; and as discussed earlier, this induces a surface defect that can initiate fatigue failure. Recently, it has been suggested that all devices should have a unique bar-code indelibly marked too; the number of defects has suddenly increased by the number of lines on the bar-code. Therefore, this paper examines, through a controlled case study, whether marking has any effect on fatigue life and whether the position and effects of marking on an implantable medical device are worthy of further investigation.

## 2. Fatigue and Component Failure

**Figure 1:**
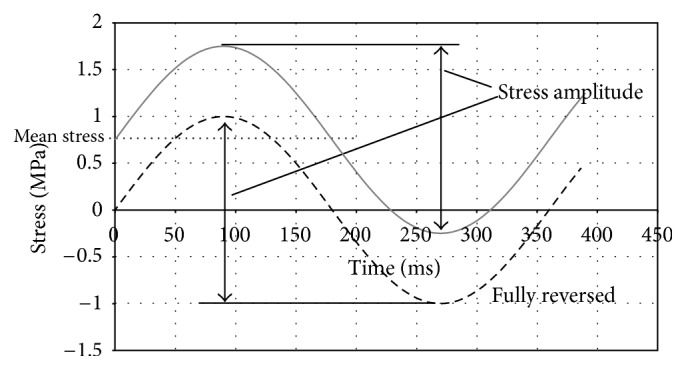
Simplified cyclic stress illustration (Δ*σ* = 2 MPa : *f* = 1.4 Hz).

**Figure 2:**
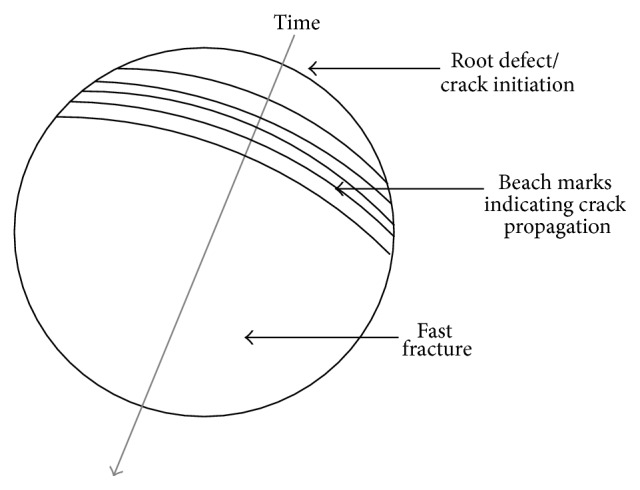
Representation of a typical fatigue failure.

Fatigue failure is a well-known phenomenon, but it is worth describing the basics at this point to facilitate later discussions. It occurs in components subject to cyclic loading. The loading may cycle between compression and tension (so-called full reversal), or it can cycle between low and high values of tensile loads (Figure 1). The variation between the maxima and minima is called the stress amplitude, and it is this value that dictates the component's fatigue life. The component's existence is one of the constantly changing stresses, and this can lead to crack propagation. Normally, an initial defect will act as the source of the crack (hence, the mirror finish is described earlier). As time goes on, the crack will grow in length, normally in discernible steps (Figure 2). At some stage, the crack is so large that the component can no longer withstand the loads imparted in it, and it will suddenly fail (often by fast fracture). Thus, a fatigue failure has three, distinct, and observable regions: the point of crack initiation, a region of crack growth (highlighted by “beach marks”), and finally a region of fast fracture. 

For components that are easily visible, crack propagation can be investigated and observed. In highly critical areas of engineering, this is conducted using nondestructive testing techniques (NDTs). However, implants in the human body do not avail themselves to gamma irradiation for crack detection, nor are they easily visible for inspection. Hence, implants must be designed to ensure that fatigue failure is minimized; and hence, they are routinely polished to a mirror finish. But they are then subjected to a defect in the form of laser marking. Recently, it has been suggested that class II (and higher) devices should be indelibly marked with a unique identifying bar code and increasing the number of defects by the number of lines in the bar-code.

## 3. Does a Laser Mark Constitute a Root Defect?

Before this question can be answered, the concept of a root defect must be evaluated. In fact, there are two concepts that are hidden within: the first is crack depth; the second is stress concentration. 

**Figure 3:**
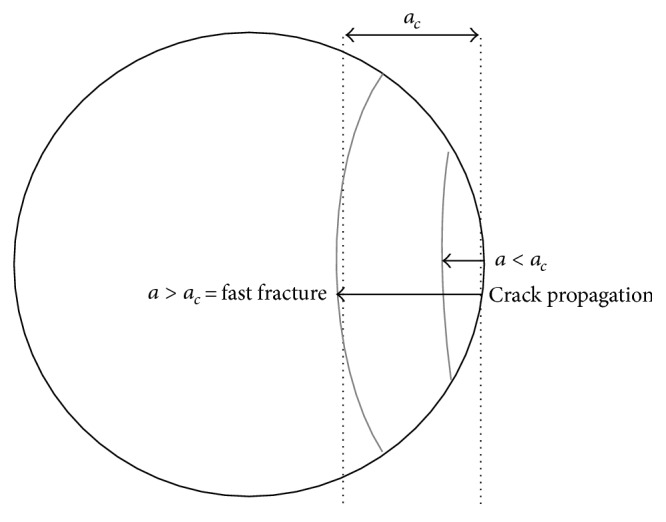
Critical crack length for fast fracture.

In relation to crack depth, this relates to the remaining life of the component and has two main issues. The first issue is critical crack length. This relates to the size of crack that leads to fast fracture of the component (the ultimate failure mode) and is determined by the fracture toughness of the material. For most materials, the critical crack length can be determined using [6]:

(1) $$\begin{array}{c} K_{c}=\sigma \sqrt{\pi a} \end{array}$$

or

(2) $$\begin{array}{c} a_{c}=\frac{1}{\pi }{\left[\frac{K_{c}}{\sigma }\right]}^{2}, \end{array}$$

where *K*_*c*_ is a material constant (fracture toughness) and *σ* is the applied stress. If the depth of the root defect is greater than *a*_*c*_, then the component will fail due to fast fracture. The common process is that the root defect is much smaller than *a*_*c*_ but, through fatigue and crack propagation, the crack finally achieves a value near to *a*_*c*_ and the component fails [6]. This is illustrated in Figure 3.

**Figure 4:**
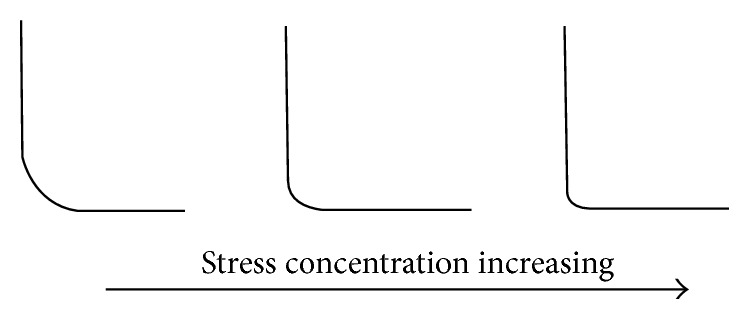
Illustration of relationship between sharp corners and stress concentration.

Stress concentrations are associated with the shape of the defect. In general, sharper defects cause greater concentrations of stress. Peterson's stress concentration factors [7] illustrate that concentration factors of 3 are highly plausible (this means that the actual stress at the root is 3 times the average stress) and the stress concentration factor is never less than 1. Hence, this means that the stress amplitude discussed previously can be much greater than initial estimates, thereby reducing fatigue life considerably. It is, therefore, a commonplace to insert a radius in a corner to alleviate stress concentrations. (as illustrated in Figure 4).

**Figure 5:**
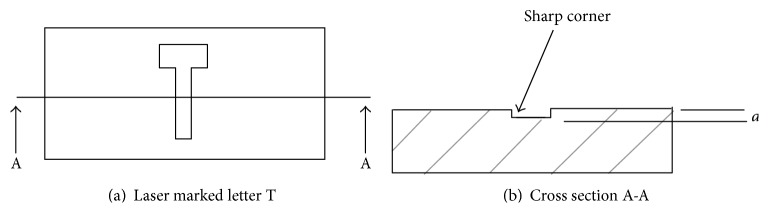
Representation of laser marking creating a defect.

The obvious question is: what does this have to do with laser marking? As previously presented, the process of marking can result in material removal. This is, in effect, the same as producing a small crack. The depth of the crack will depend on the power of the laser, the duration of exposure to the beam, and the number of passes. However, unlike physical machining, one has no control of the radius at the corners. This is illustrated in Figure 5.

**Figure 6:**
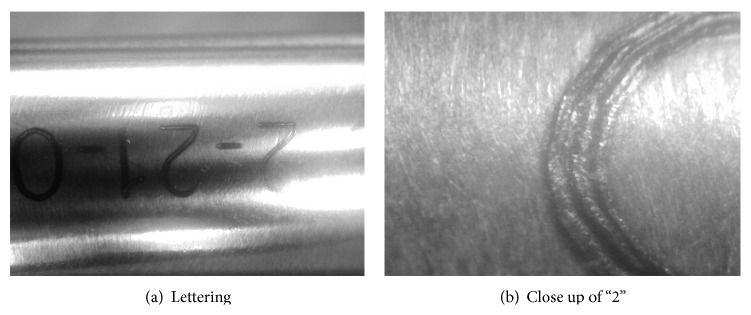
Laser marked lettering of a component.

Whilst the illustration in Figure 5 is theoretically plausible, this does not represent factual existence.  Figure 6 illustrates the marking of a typical titanium (Ti 6AL 4V) medical component.  Figure 6(a) illustrates the marking, and Figure 6(b) illustrates a magnification of the lettering.

Figure 6 clearly demonstrates that the laser marked lettering can be considered to be a defect. In the case of number “1”, the transverse nature of the letter makes this highly dangerous. Qi et al. [8] examined the effect of frequency on the depth of laser marking of stainless steels. They demonstrated that 25 *μ*m is achievable with ease. This exceeds the defect size of 0.1–0.8 *μ*m expected from electropolishing or even the 1.6 *μ*m expected from simple grinding. A selection of components from a variety of manufacturers had the laser marking assessed using a Mitutoyo surface roughness analyzer. As expected, there was a great deal of variability due to the variability of substrate and laser etching methodology. However, these initial investigations illustrated that laser marking can exceed 25 *μ*m with ease.

It is, therefore, argued that laser marking does constitute a defect.

## 4. Case Study to Determine Effect

**Figure 7:**
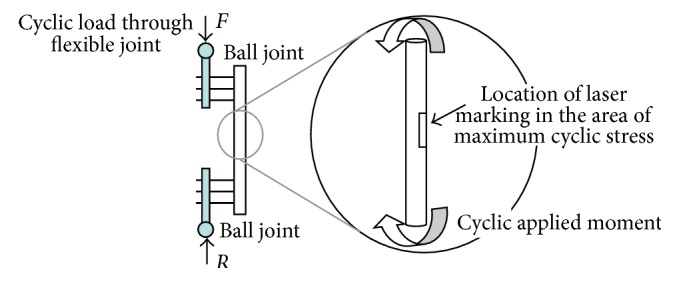
Schematic of experiment.

For the purposes of this case study, a monolateral fixation is assumed (as illustrated in Figure 7). Under normal, static, body weight, the applied force *F* would be in the region of 900 N (clearly dependent on body mass and gait—when walking the peak load would increase to 1.1 kN). This applied force creates an overall value compressive stress, but the stress in the system is dominated by the bending stress generated by the applied moment. 

### 4.1. Fast Fracture

The component is made from Ti 6Al 4V, then its fracture toughness is, approximately, *K*_*c*_ = 75 MPa m^1/2^ [9]. Further, assuming the maximum stress is 600 MPa, then the critical crack length is given (from (2)) as

(3) $$\begin{array}{c} a_{c}=\frac{1}{\pi }{\left[\frac{75}{600}\right]}^{2}=0.0049\;\text{m}. \end{array}$$

Hence, the deformity of 25 *μ*m is not a cause for concern.

### 4.2. Fatigue Life

From Basquin's law [6]

(4) $$\begin{array}{c} \Delta \sigma =C_{1}{N_{f}}^{B} \end{array}$$

and the data for Ti 6Al 4V from Casavola et al. [10] where *B* = −0.09 and *C*_1_ = 1068, the uncracked fatigue life for the component can be estimated. Assuming that the stress amplitude is 340 MPa, hence, the number of cycles to failure is

(5) $$\begin{array}{c} 340\;=\;1068N_{f}^{-0.09},\\ N_{f}=\;333.6\times 10^{3}\;\text{cycles}, \end{array}$$

a figure that would be slightly concerning for a long-term implant but not for something that is going to be used in short term. Let us now consider the effect of the defect. Using the Paris' law fatigue crack growth model [6]:

(6) $$\begin{array}{c} \frac{da}{dN}=C{\left(\Delta K\right)}^{n}, \end{array}$$

where the constants *C* and *n* are material dependent, Ghidini et al. [11] present fatigue data for Ti 6AL 4V for space applications and their data suggests that *C* = 6 × 10^−13^ and *n* = 4. This means that (6) can be rearranged and integrated to yield the number of cycles for a crack to grow from one size to another:

(7) $$\begin{array}{c} N=\frac{1}{6.13\times 10^{-4}{\left(\Delta \sigma \right)}^{4}\pi^{2}}\left\{\frac{1}{a_{1}}-\frac{1}{a_{2}}\right\}. \end{array}$$

The starting crack size *a*_1_ = 0.0025 mm. For this to grow to the critical crack size *a*_2_ = 4.9 mm, the number of cycles can be estimated as

(8) $$\begin{array}{c} N=\frac{1}{6\times 10^{-13}{\left(340\right)}^{4}\pi^{2}}\left\{\frac{1}{0.000025}-\frac{1}{0.0049}\right\},\\ N=503\times 10^{3}\;\text{cycles}. \end{array}$$

Table 1 presents fatigue lives when the initial depths are 25, 50, and 75 *μ*m, respectively. It demonstrates that setting the maximum marking depth to be 25 mm would create no further reduction in fatigue life compared to that of an unmarked component.

Interestingly use of this calculation enables the designer to determine the maximum marking depth. The other values all reduce fatigue life by up to 50% compared with the uncracked component. This estimate has not allowed for the effect of stress concentrations nor for the effect of increasing stress as crack length increases. Clearly, increasing the starting depth of the deformity reduces the fatigue life further.

Once again, this suggests that laser marking does constitute a deformity that has the ability to reduce fatigue life.

## 5. Experimental Investigation

A controlled experiment was conducted on a simple component manufactured from Ti6AL4V. The component was a single piece monolateral external fixator used in the treatment of distal tibial fractures. Both components were of identical manufacture, but one was laser marked and one was not. Because the design of the component is mechanically simple, it was easy to ascertain the location of maximum stress, and as a consequence, the laser marking was placed where its effect was likely to be the greatest. Figure 7 illustrates a schematic of the experiment.

The component was cyclically loaded from 0 to 900 N using a sine function of frequency 3 Hz, causing a cyclic stress amplitude of 340 MN/m^2^. Using Basquin's law and the data presented earlier, the estimated life is 333,192 cycles. The actual experimental results are illustrated in Table 2.

**Figure 8:**
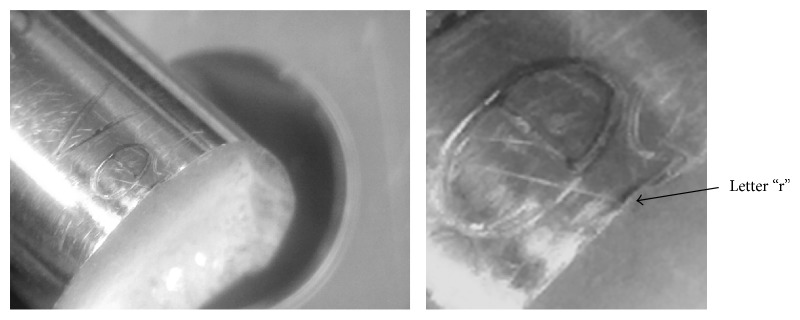
Magnified view of the root.

Although one could argue that the results may be misleading, an investigation of the root source of the crack demonstrates that it was the laser marking that gave rise to the fatigue crack (Figure 8). This further suggests that laser marking can reduce fatigue life.

## 6. The Impact on Medical Device Design

Most engineers would not be surprised by the results illustrated above. It is well known that the addition of a defect on a component reduces its fatigue life. So, why is this fact not well documented in the literature associated with medical implants? Why do we find references associated with space applications and not with medical applications? Why is it that the position of laser marking is often left to chance rather than to design? The results of this paper suggest that the placement of laser marking is highly critical. Select the wrong position and trouble, in the form of premature failure, will follow. The main issue, however, is not selecting a position but actually determining where marking cannot be placed. For example, minimally invasive plates are becoming more widespread in orthopaedic trauma. Being minimally invasive means that they are by definition slender. Which, in turn, means they are subject to high stresses. However, design on the CAD system does not fully represent how the loading actually occurs in real life. The placement of the plate and the position of fixings are, almost, random, but there will be a worst-case scenario that does not assume perfect placement. It is, therefore, incumbent on the designer to ensure that they imagine worst case scenarios and determine the location of maximum stresses. Only in this way can the location of laser marking be determined. Almost certainly, the location of the laser marking cannot be left to the discretion of the marker, nor to the discretion of the draftsman; the location where marking cannot be made must be analysed and specified in the design process. It is almost certain that it is incumbent on the design engineer to specify the position where markings cannot be made.

The work also demonstrated that there is a lack of papers/specific research in this important area. Stress corrosion has not been considered, nor has there been a full analysis to determine if pulse rate can be used to mitigate effects or whether different marking techniques could be adopted or developed. It would seem that the effect of laser marking has been treated in a “matter of fact” way when its effects can be disastrous (as in [3]). It is not possible to, simply, extract data from the aerospace industries (where loading patterns can be prescribed) and assume that this must be the case for medical devices (where the loading regime is not prescribed). Certainly, the first important study is to determine suitable marking depths for critical materials (to ensure legibility) and for clinical evaluations to consider this depth in the analysis. Certainly, future clinical evaluations should cover marking location in the risk analysis. For long term implants, more important are the consequences of stress corrosion. 

It is suggested, due to the lack of research papers in this field, that further research should be conducted on the effects of laser marking on the life of implantable and nonimplantable medical devices.

## 7. Conclusions

The limited study presented in this paper suggests that laser marking can be deleterious to the fatigue life of a medical device. The use of laser marking is widespread and is, probably, the only commercially viable permanent marking system available. Hence, it is incumbent on medical device designers to document and justify the position of laser marking to ensure that it does not, by pure chance or mishap, become located in a region where high cyclic stresses are experienced. This may not be as simple as it first seems as many implantable medical components may not, actually, be used in the exact configuration they were designed at. This is not misused by the surgeon or clinician; this is a fact which is wholly due to the variability associated with humans and their range of potential ailments. This is particularly true in orthopaedic trauma where, for example, the range of fracture patterns for one particular device can be highly variable.

## Figures and Tables

**Table 1:** Comparison between fatigue life and initial marking depth.

Initial depth	*a*_1_ = 25 *μ*m	*a*_1_ = 50 *μ*m	*a*_1_ = 75 *μ*m	*a*_1_ = 37.6 *μ*m
Life (cycles)	503 × 10^3^	250 × 10^3^	165 × 10^3^	333.6 × 10^3^
Reduction in life	N/A	25%	50%	0%

**Table 2:** Experimental fatigue lives.

Theory	Unmarked specimen	Laser marked specimen	Reduction
333,617	315,789	99,745	70%

## Notations

*A*: Paris' law constant
*a*: Crack length (m)
*a*_*c*_: Critical crack length (m)
*B*: Basquin's law constant
*C*_1_: Basquin's law constant
*K*: Stress intensity Factor (MN m^−3/2^)
*K*_*c*_: Fracture toughness (MN m^−3/2^)
*M*: Paris' law constant
*N*: Number of cycles
*N*_*f*_: Number of cycles to failure
*σ*: Stress (MN m^−2^).
